# Alkaline Phosphatase and Infantile GM1 Gangliosidosis: A Simple Biomarker for a Complex Disease?

**DOI:** 10.1002/jmd2.70075

**Published:** 2026-03-05

**Authors:** Laura Fiori, Massimiliano Turzi, Veronica Maria Tagi, Laura Asnaghi, Eleonora Bonaventura, Davide Tonduti, Luigina Spaccini, Laura Assunta Saielli, Chiara Montanari, Francesca Cairello, Savina Mannarino, Matilde Ferrario, Alessandra Del Longo, Marcello Napolitano, Andrea Righini, Michela Semeraro, Anna Venerando, Martina Miceli, Elvira Verduci, Gianvincenzo Zuccotti

**Affiliations:** ^1^ Department of Pediatrics Vittore Buzzi Children's Hospital Milan Italy; ^2^ Metabolic Diseases Unit, Department of Pediatrics Vittore Buzzi Children's Hospital Milan Italy; ^3^ Department of Biomedical and Clinical Science University of Milan Milan Italy; ^4^ COALA (Center for Diagnosis and Treatment of Leukodystrophies), unit of Pediatric Neurology, Vittore Buzzi Children's Hospital Milan Italy; ^5^ Clinical Genetics Unit, Department of Obstetrics and Gynecology, Buzzi Children's Hospital University of Milan Milan Italy; ^6^ Center of Functional Genomics and Rare Diseases Vittore Buzzi Children's Hospital Milan Italy; ^7^ Pediatric and Pediatric Emergency Unit, Pediatric Cardiology Service, the Children Hospital, AO SS Antonio e Biagio e C. Arrigo Alessandria Italy; ^8^ Pediatric Cardiology Unit, Department of Pediatric Buzzi Children's Hospital Milan Italy; ^9^ Pediatric Ophthalmology Unit, ASST Grande Ospedale Metropolitano Niguarda Milan Italy; ^10^ Department of Radiology and Neuroradiology Vittore Buzzi Children's Hospital, University of Milan Milan Italy; ^11^ Division of Metabolism and Research Unit of Metabolic Biochemistry Bambino Gesù Children's Hospital, IRCCS Rome Italy; ^12^ Unit of Medical Genetics and Neurogenetics Fondazione IRCCS Istituto Neurologico Carlo Besta Milan Italy; ^13^ Medical Genetics Unit, ASST Santi Paolo e Carlo Milan Italy; ^14^ Department of Health Sciences University of Milan Milan Italy

**Keywords:** alkaline phosphatase, biomarker, diagnosis, gangliosidosis, GM1, infantile

## Abstract

GM1 gangliosidosis is a lysosomal storage disease (LSD) caused by β‐galactosidase deficiency, characterized by the accumulation of gangliosides in various tissues. Among different GM1 forms (infantile form, late‐infantile and juvenile form, and late‐onset form), the infantile form is the most severe: despite an early clinical onset with rapid neurodegeneration, coarse face, abdominal visceromegaly and skeletal abnormalities, the diagnosis is usually delayed, given the lack of recognized early disease‐specific markers. We report the case of a newborn presenting with mild edema of hands and feet, mild transient hypoalbuminemia and isolated hyperphosphatasemia at three weeks of life. The first cardiological evaluation showed mild mitral regurgitation. Despite the absence of neurological symptoms, organomegaly, or a coarse face, the turgid consistency of the limbs, together with mitral regurgitation and persistent hyperphosphatasemia, led to multiorgan investigations with discovery of bilateral cherry‐red spots and a beak‐shaped lumbar vertebra. The cardiological follow‐up revealed a dysplastic mitral valve. In the suspicion of a lysosomal disease, biochemical investigations were planned. An altered profile of urinary oligosaccharides, along with low β‐galactosidase activity in leukocytes, led to the diagnosis of infantile GM1 gangliosidosis at 3 months of age. The *GLB1* gene analysis confirmed the diagnosis. Genetic testing for GLB1 should be considered in cases of persistent hyperphosphatemia, especially if it is associated with any other clinical indicator of GM1, such as limb edema.

## Summary

- Clinicians should consider GM1 gangliosidosis in newborns and infants with unexplained hyperphosphatasemia and any other clinical indicator of GM1, such as edema of the limbs, even in the absence of other macroscopic clinical signs of this disease.
- Identification of early, albeit nonspecific, biochemical markers may enable timely diagnostic evaluation, including GLB1 genetic testing, and facilitate appropriate patient management.

## 1 Introduction

GM1 gangliosidosis is a rare autosomal recessive lysosomal storage disease (LSD) caused by a deficiency of the enzyme *β*‐galactosidase (β‐gal, EC number 3.2.1.23) [1]. This enzyme, encoded by the *GLB1* gene (OMIM number 611458), located on chromosome 3, allows cleavage of the β‐1,4‐linked galactose terminal from the cell membrane glycosphingolipid called ganglioside, as well as from other glycan moieties.

A deficiency in β‐gal activity leads to the accumulation of toxic gangliosides in several tissues, particularly nervous and bone tissues, liver, spleen, and heart [1, 2].

There are four types of GM1 described. Infantile or type 1 GM1 is characterized by early‐onset with rapid neurodegeneration. Type 2 GM1 consists of late‐infantile and juvenile forms, affecting preschool and school‐aged children with progressive neurodegeneration, which is later associated with cardiac valvular disease in the juvenile‐onset form. Type 3 GM1, the a late onset/adult form, is characterized by cerebellar ataxia and progressive dementia. Despite signs and symptoms of type 1 GM1 typically appearing within 6 months of age, the average age at diagnosis is around 8 months, as the elevated liver enzymes observed at presentation in most of the patients represent nonspecific biochemical data [3]. Liver enzymes are usually elevated at presentation in most patients but unfortunately represent nonspecific biochemical data.

Elevated alkaline phosphatase (ALP) levels reflect increased synthesis or release of ALP isoenzymes, mainly originating from the liver and bone. The most common mechanisms include cholestatic or infiltrative liver diseases and increased bone turnover associated with osteoblastic activity [4, 5]. In pediatric patients, physiological bone growth represents a frequent cause of ALP elevation, although persistent or unexplained hyperphosphatasemia warrants further investigation [5].

We describe the case of an early diagnosis of GM1 infantile form that presented with limbs turgidity, cardiac abnormalities, mild hypoalbuminemia, and persistent elevations of ALP as unique biochemically relevant parameters since the first weeks of life.

## 2 Methods

The analysis of urinary oligosaccharides was performed with a UHPLC–MS/MS method, with MRM acquisition of target oligosaccharides transitions in positive and negative modes, switching the polarity within a single run. The chromatographic run was < 30 min.

The quantification of total urinary glycosaminoglycans (GAGs) was performed with a colorimetric method that involves using a dye, 1,9‐dimethylmethylene blue (DMB), which binds to GAGs, causing a color change that can be measured spectrophotometrically.

The β‐galactosidase activity was measured in leukocytes, isolated from ethylenediamine tetra‐acetic acid‐treated blood by hypotonic lysis of erythrocytes and subsequently frozen. The enzyme activity was determined by incubating leukocytes with 4‐methylumbelliferyl‐β‐D‐galactopyranoside, following the method by Ho M. W. et al. [6]. The reactions were terminated by the addition of Na_2_CO_3_‐NaHCO_3_ buffer at pH 10.7, and the fluorescence of 4‐methylumbelliferone was measured using an LS55 Fluorescence Spectrometer (PerkinElmer) with excitation at 385 nm and emission at 450 nm.

Analysis of the *GLB1* gene was performed by the Medical Genetics Unit, ASST Santi Paolo e Carlo, Milan, Italy, using next‐generation sequencing (NGS).

For further diagnostic evaluation, the patient underwent brain magnetic resonance imaging (MRI) to assess possible pathological findings, abdominal ultrasound to exclude organomegaly, and a total body X‐ray to detect any skeletal deformities. Additionally, echocardiography was performed to follow up on the previously noted cardiac findings, and optical computerized tomography (OCT) was carried out to investigate potential ocular abnormalities.

## 3 Case Report

A female infant was born at term to a bichorial biamniotic pregnancy from related parents (first‐degree cousins). She was the fourth born with 2 older siblings and a twin male brother, all in good health. Prenatal ultrasounds were reported as normal.

The patient's birth weight was at the 5th percentile according to gestational age, consistent with low birth weight. Apgar scores at 1 min and 5 min were 9. Except for phototherapy due to neonatal jaundice, no complications were registered during the neonatal period. The baby was discharged from the hospital at 5 days of life on formula feeding.

At 14 days of life, the newborn was taken to the Emergency Department with mild edema in hands and feet and left atrial enlargement with mitral valve regurgitation. Blood investigations revealed hypoalbuminemia (albumin 1.8 mg/dL, nv 3.5–5 mg/dL) which normalized spontaneously in a couple of days, together with the edema. The albumin‐corrected calcium level (9.8 mg/dL, nv 8.5–10.5) and the phosphate level (6.4 mg/dL, nv 4.8–8.4) were within the normal range.

At the following cardiological evaluation at 30 days of life, worsening of mitral regurgitation with left chamber overload at echocardiography was disclosed and a low dose furosemide treatment (0.5 mg/Kg/day) was started. Subsequently, peripheral edema at hands and feet appeared again: the drug dosage was increased to 1.5 mg/Kg/day and the patient was referred to our Center for further investigations at 3 months of life. She presented in good general condition with normal vital parameters, properly formula fed with normal suction, without vomiting or diarrhea, and showing good weight gain. Mild turgid consistency of the limbs (forearms, legs and thighs) was present at palpation. Pulmonary and abdominal evaluations were normal. Neurological examination was also normal. No facial dysmorphisms were evident. Upon arrival, first‐line biochemical blood tests were performed, revealing hyperphosphatasemia, which was confirmed at two following evaluations (ALP: 1640–1590–1744 U/L, nv 130–518 U/L), with remaining bone function tests and transaminases within normal values. Persistent mild hypoalbuminemia (2.9 mg/dL) was detected too. Abdominal ultrasound resulted normal. A nephrological consultation excluded the renal origin for hypoalbuminemia. Despite isolated hyperphosphatasemia usually being attributed to a benign condition called transient hyperphosphatasemia [7], the turgid consistency of the limbs and the hyperphosphatasemia, already described in some GM1 patients [8, 9, 10, 11], led to hypothesize a LSD.

**FIGURE 1:**
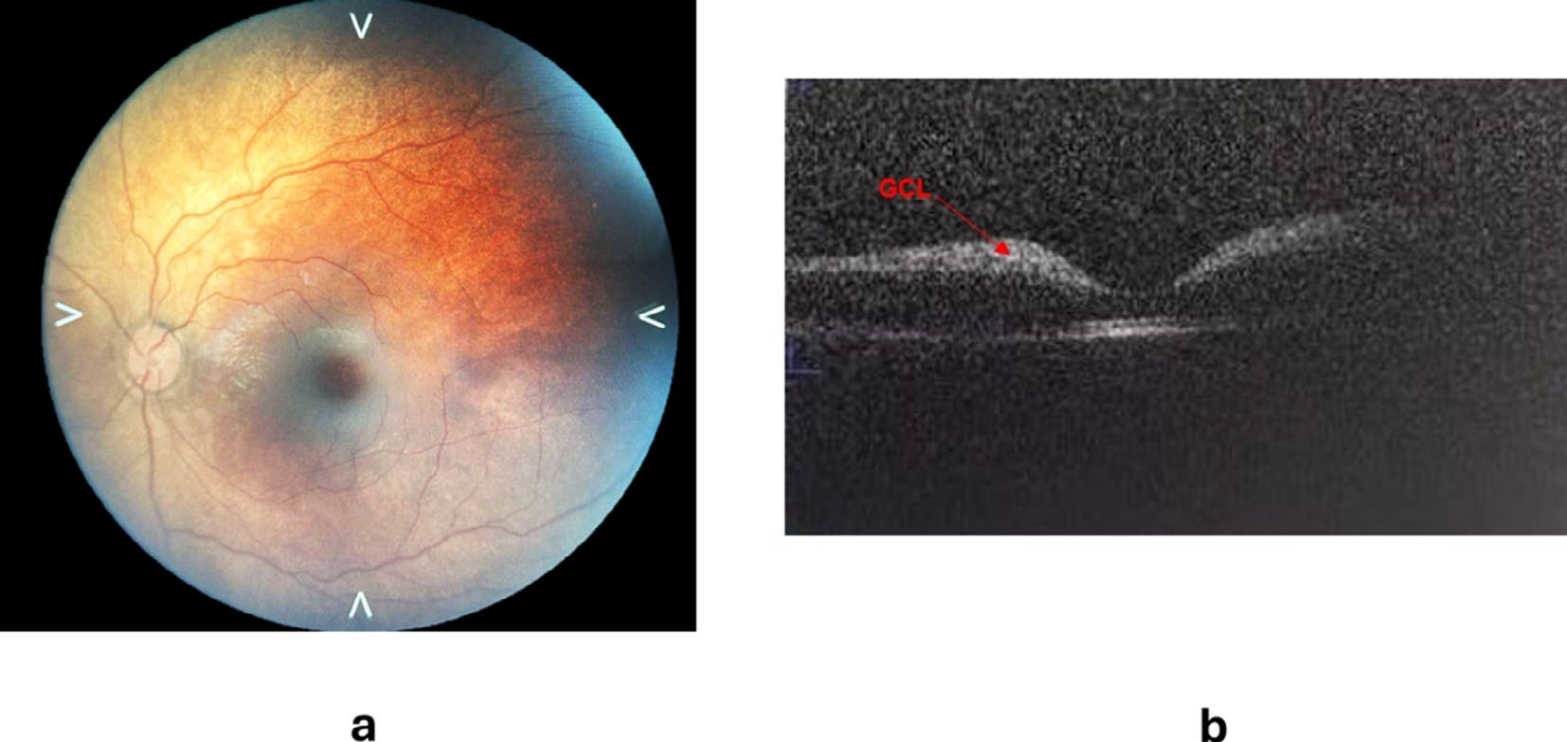
Cherry‐red spot (right eye picture) (a) and increased reflectivity in the ganglion cell layer (GCL) on optical computerized tomography (OCT) (arrow) (b).

**FIGURE 2:**
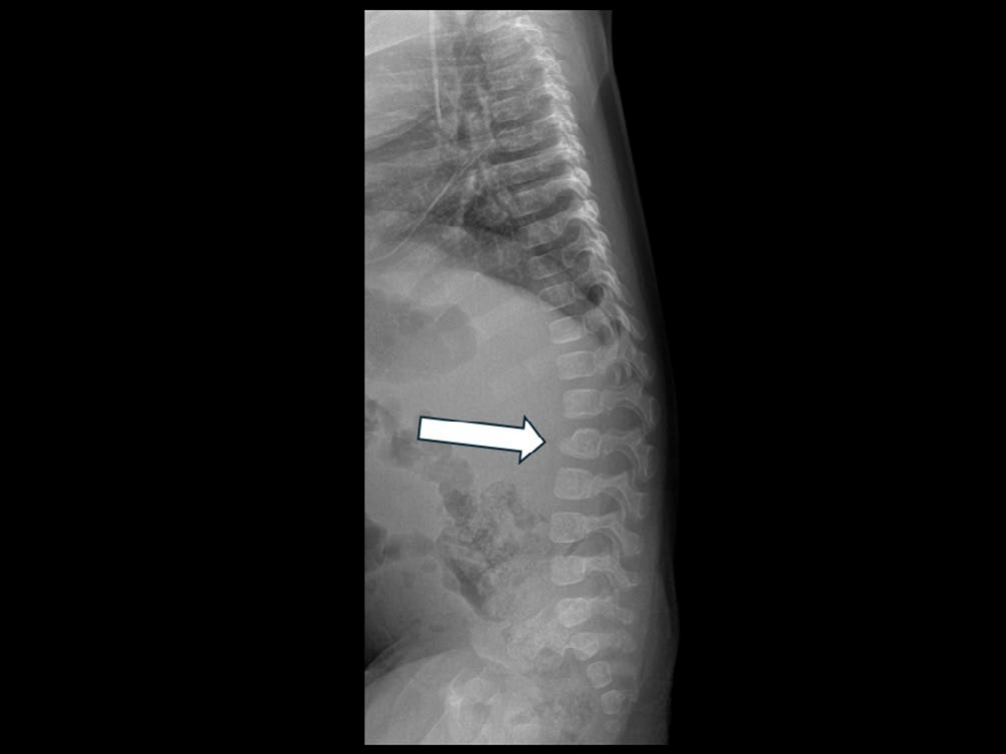
Lateral X‐ray projection film of the spine showing beak‐like shaped L2 body (arrow).

**FIGURE 3:**
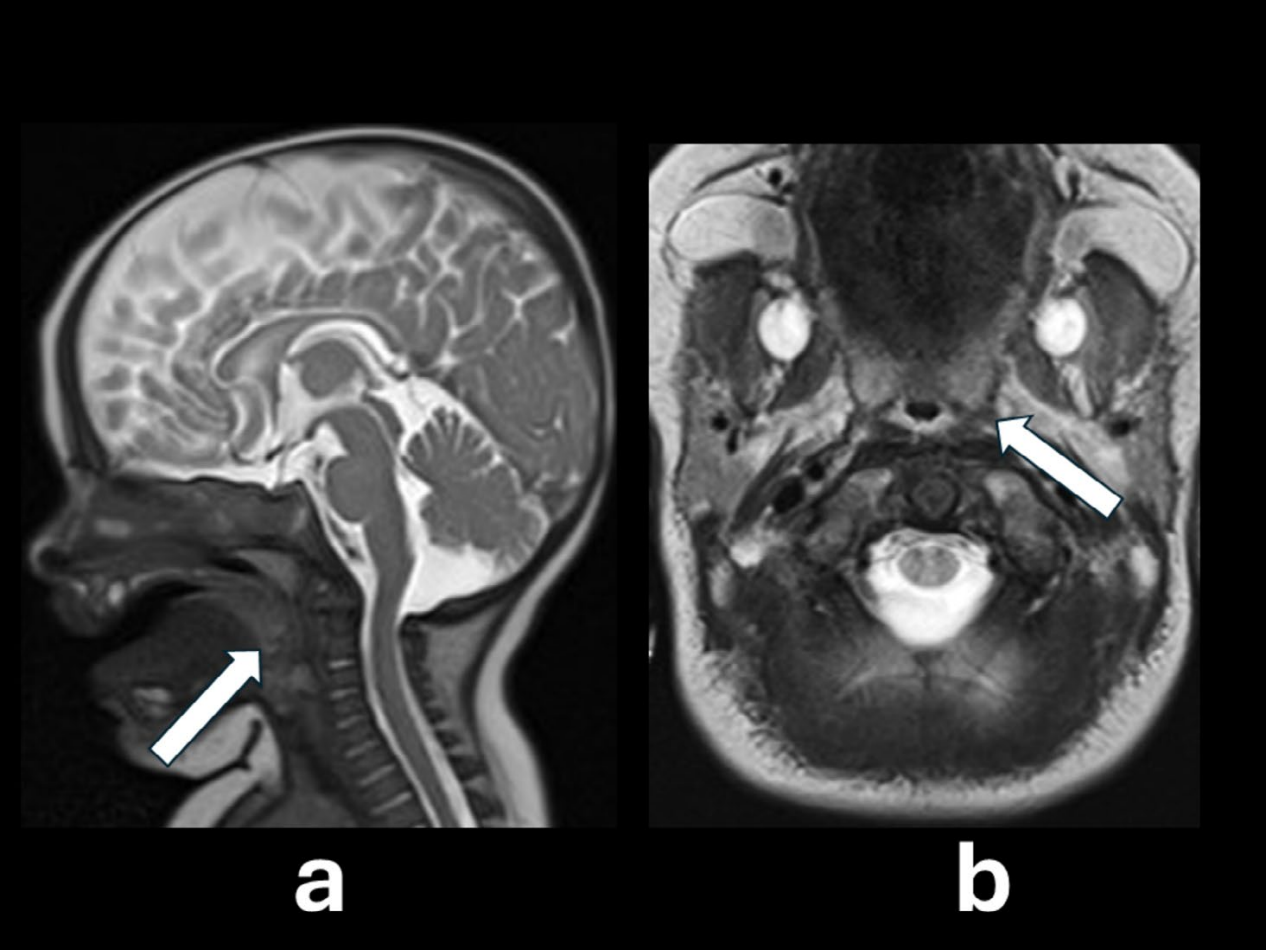
Sagittal (a) and axial (b) T2‐weighted MR‐imaging sections showing a mild frontal cerebrospinal fluid space enlargement and nonspecific increase in the conspicuity of the concentric soft tissue around the oral and rhinopharynx, which appear moderately stenotic (arrows).

The ophthalmological examination revealed a bilateral cherry red spot, deeply investigated by OCT on an awake baby. The OCT showed increased reflectivity in the ganglion cell layer (GCL) and the boundaries between the hyperreflective and normal regions were not clear [Figure 1]. The total body X‐Ray revealed a dysmorphic beak‐like shape of the second lumbar vertebra [Figure 2]. Brain MRI showed mild frontal cerebrospinal fluid space enlargement and nonspecific increase in conspicuity of the concentric soft tissue around the oral and rhinopharynx, which appeared moderately stenotic [Figure 3]. Abdominal ultrasound revealed a liver and spleen of normal echogenicity and size appropriate for the patient's age. Auditory evoked potentials were normal.

Echocardiography was repeated and showed a dysplastic, thick mitral valve with regurgitation and right atrial enlargement. Treatment with captopril at a dose of 0.5 mg/kg/die was then introduced with good cardiological response.

Metabolic investigations were performed. Urinary GAGs were mildly elevated on two occasions (211 and 315 μg/mg urinary creatinine, respectively, nv 20–200), probably due to urinary creatinine below the age‐adjusted reference range, normal in the third sample (45.5 mg/mmol urinary creatinine, nv < 52), performed in a different laboratory; chitotriosidase on dried blood spot was normal (96 micM/h/L, nv 8–121). We evaluated the urinary oligosaccharide profile on DBS, which was compatible with GM1, and β‐galactosidase activity in leukocytes, which was also consistent with GM1 (11.2 nmol/h/mg; nv: 84–292). Upon *GLB1* gene analysis, the patient was found to be homozygous for a pathogenic variant (class 5) in exon 2: NM_000404.4:c.176G>A p.(Arg59His).

As the GLB1 gene analysis, the patient was found to be homozygous for a pathogenic variant (class 5) in exon 2: NM_000404.4:c.176G>A p.(Arg59His).

Our patient is now 10 months old. From 6 months of age, she experienced a progressive neurological deterioration with rapid regression of motor milestones, followed by the onset of dysphagia at 8 months.

## 4 Discussion

We report an early diagnosis of infantile GM1 gangliosidosis, presenting with a turgid consistency of the limbs, mitral valve regurgitation, and marked, persistent, isolated hyperphosphatasemia.

The infantile form of GM1 is the most severe of the three GM1 gangliosidosis types. Symptoms onset is usually within 6 months of age and the natural history of the disease leads to early death in most patients [2, 12].

Many clinical signs overlap with the infantile GM2 gangliosidosis (Tay‐Sachs disease, Sandhoff disease). Jarnes Utz JR et al. described the natural history of infantile gangliosidoses, including both GM1 and GM2 patients. Among 8 patients suffering from infantile GM1 gangliosidosis, the median age of onset of the first noted symptom was 1.5 months. The most common clinical signs in both infantile gangliosidoses are the onset of hypotonia within the first 6 months of life, excessive oral and respiratory secretions, dysphagia, gastroesophageal reflux, and constipation. Patients with infantile GM1 were less likely to gain head control at any time, and most patients had seizure disorders by 18 months of age [12]. Vertebral beaking at lateral X‐ray evaluation and cardiomegaly at cardiological investigations were present in some infantile GM1 patients but not in GM2 patients. In GM1 gangliosidosis, vertebral beaking most commonly involves the thoracolumbar spine, particularly the lumbar vertebrae; however, the reason for this preferential lumbar involvement remains unclear [13]. In comparison to mucopolysaccharidoses, where bone involvement is more severe because stored glycosaminoglycans directly disrupt cartilage and bone homeostasis, leading to progressive skeletal dysplasia, growth plate destruction, and chronic inflammation [14], the accumulation of GM1 ganglioside predominantly affects neural tissue, with skeletal involvement being less pronounced and generally milder [13].

The severity of the infantile form of GM1 gangliosidosis versus the late‐infantile juvenile and late onset types is described by Arash‐Kaps L et al. in a cohort of German patients [1]. Visceromegaly, cardiomyopathy, and hepatosplenomegaly are typical symptoms of patients with the infantile form, along with facial dysmorphism and neurological impairment. Muscular hypotonia and eating difficulties are usually precocious, as already described, together with seizures. Radiologic evidence of multiplex dysostosis, especially spinal deformity, is frequently a precocious sign also described by these authors [1]. Regarding the biochemical characteristics of all three types of GM1 gangliosidosis, the authors observed an increase in the liver enzyme aspartate‐aminotransferase (ASAT) in most patients and pointed it out as a biochemically relevant red flag, together with chitotriosidase and urinary oligosaccharides. ALP is not mentioned among biochemical investigations performed in these patients.

Even if most patients with infantile and late‐infantile GM1 gangliosidosis are reported to have hepatosplenomegaly [2] and increased chitotriosidase activity [1], these were not present in our child. However, her neurological condition has markedly worsened since 6 months of age, resulting in a very severe clinical presentation.

Some authors described nonimmune hydrops fetalis and cardiac disease detected prenatally in some infantile GM1 patients. Therefore, this clinical sign should be deeply investigated in the suspect of a LSD when discovered, as described in literature [15]. Limb edema in the postnatal period has been described in some newborns with infantile GM1 gangliosidosis, together with hypoalbuminemia among biochemical investigations. Authors report this biochemical sign in both untreated human GM1 patients and in GM1 cats, describing lower levels in more severely affected infants [16]. ALP too is described by these authors, with statistically higher levels in infantile GM1 patients than in late‐infantile and juvenile patients. Anyway, authors ascribe higher ALP levels to a loss of bone mass secondary to impaired walking in this group of patients, but this etiopathogenetic hypothesis can only be valid for patients older than the average age at which walking ability begins.

Elevated ALP levels have also been reported in other LSDs, particularly in those with skeletal involvement such as mucopolysaccharidoses and Gaucher disease [4]. This finding has been attributed to the accumulation of non‐degraded substrates within the lysosomes of bone and cartilage cells. This accumulation interferes with normal cellular processes, resulting in defective bone mineralization, altered growth plate structure, and increased osteoblastic activity. Consequently, skeletal dysplasia and deformities promote enhanced production and release of bone‐specific ALP into the bloodstream [4, 17]. However, the degree of ALP elevation varies among different LSDs and individual patients, and is not universally present in all cases [4].

GM1 diagnosis is usually delayed in time. In fact, although signs arise and evolve relatively rapidly within a few months, they are often overlooked during clinical evaluations or brought to the attention of single‐organ specialists, as usually happens in rare diseases before diagnosis.

Isolated hyperphosphatasemia in medicine is usually attributed to a benign condition called transient hyperphosphatasemia, which usually does not raise clinical concerns [6] but may be a specific biochemical sign of infantile GM1, as seems to emerge not only from our clinical case but even from previous publications [8, 9, 10, 11].

Caciotti A et al. [10] described a child who presented with limb edema and an increase in plasma ALP at 4 months of age, initially ascribed to a perinatal clavicular fracture. No cardiac dysfunction or cardiac structural abnormalities or valve defects were found at that age, while X‐ray showed bone demineralization and early rachitic changes. Psychomotor development in the child was normal until 1 year of age, while mild coarseness of the face and hepatosplenomegaly became relevant in the previous months revealing a multisystemic involvement.

The isoforms of ALP have been recently described by Menkovic I in a GM1 patient, who showed high ALP levels since the age of 16 days [11]. In this patient a diagnosis of cardiac abnormalities was formulated by prenatal ultrasound, showing a dilated right atrium and ventricle, tricuspid valve regurgitation, and a tortuous ductal arch, confirmed after birth. No dysmorphic features were noted. Due to brain intraventricular hemorrhage and respiratory insufficiency soon after birth, he required supplemental oxygen and intensive treatment, while biochemical exams revealed elevated ALP. Transient hyperphosphatasemia in infancy was initially suggested but not confirmed with other bone mineral markers within reference limits. In this patient most of the increase in ALP was referred to the bone isoenzyme (73.1%), while 21.8% and 5.1% were related to gut and liver isoenzymes respectively. At the follow up at almost 3 months of age neurological impairment was registered, describing central hypotonia, head lag, and hypertonia in the extremities together with coarseness of the face. At that time anterior beaking of a few lumbar vertebral bodies and hemivertebrae of the lower cervical spine were demonstrated in the X‐ray examination. Clinical exome sequencing analysis showed two heterozygous pathogenic variants in the *GLB1* gene.

To the best of our knowledge, elevated ALP levels have not been reported in GM2 to date.

In our patient the turgid consistency of the limbs (legs, thighs, forearms) was the only clinically detectable sign in the first weeks of life. The additional presence of mitral valve insufficiency, although it was a nonspecific sign, raised suspicion for a LSD, even in the absence of a coarse face and hepatosplenomegaly. The evidence of persistent ALP increase, with normal bone mineral markers (25 OH‐D vitamin, phosphorous, calcium, parathyroid hormone) and normal transaminase, led to further deeper multiorgan investigation itself.

## 5 Conclusions

This case highlights the importance of considering GM1 gangliosidosis in the differential diagnosis of infants presenting in the first weeks of life with persistent hyperphosphatasemia, especially if edema of the limbs is present and/or a cardiac disease is found. Even if hepatosplenomegaly and/or hypertransaminasemia are not present, hyperphosphatasemia itself could be a good pre‐clinical indicator of GM1 infantile gangliosidosis in early life, supporting the recent hypothesis by Menkovic I et al. [11]. Therefore, genetic testing for *GLB1* should be taken into consideration in case of persistent hyperphosphatemia, especially if associated with any other clinical or biochemical indicator of GM1.

The differential diagnosis should include LSD in case of hyperphosphatasemia, even when initial biochemical findings appear benign or nonspecific. The concomitant presence of clinical signs in two different organs should lead to the suspicion of a systemic rare disease.

Furthermore, ALP is a biochemical investigation available and easy to do in most laboratories. Unfortunately, there are no effective treatments for GM1 gangliosidosis. Enzyme replacement therapy (ERT), enzyme enhancement therapy (EET), substrate reduction therapy (SRT) such as Miglustat, stem cell transplantation (SCT), and gene therapy have been proposed but are not therapeutically effective strategies; multiple mouse models of this disorder have been instrumental for pre‐clinical testing of multiple therapies, several of which are currently in clinical trials [3, 18].

The prognosis of infantile GM1 form is by now poor [2]. Anyway, while no curative therapies are currently approved, early diagnosis in GM1 gangliosidosis and LSD in general may allow timely enrollment in clinical trials. Furthermore, after diagnosis, knowledge of the natural history of this LSD allows for a more appropriate approach to the clinical signs and complications that arise during disease progression, leading to coordinated multidisciplinary management, and improving the quality of life of patients. Doubtless, genetic counseling for the family is crucial.

## Author Contributions

**Laura Fiori:** writing – original draft, methodology, investigation, formal analysis, data curation, conceptualization. **Massimiliano Turzi:** writing – original draft, investigation. **Veronica Maria Tagi:** writing original draft, investigation. **Laura Asnaghi:** investigation. **Davide Tonduti:** investigation, writing – review and editing. **Eleonora Bonaventura:** investigation. **Luigina Spaccini:** investigation. **Laura Assunta Saielli:** investigation. **Chiara Montanari:** investigation. **Francesca Cairello:** investigation. **Savina Mannarino:** investigation, writing – review and editing. **Matilde Ferrario:** investigation. **Alessandra Del Longo:** investigation. **Marcello Napolitano:** investigation. **Andrea Righini:** investigation. **Michela Semeraro:** investigation, methodology, data curation. **Anna Venerando:** investigation, methodology, data curation. **Martina Miceli:** investigation. **Elvira Verduci:** visualization, supervision. **Gianvincenzo Zuccotti:** visualization, supervision.

## Funding

The authors have nothing to report.

## Ethics Statement

Ethical approval was deemed not required for this case report as it involves the reporting of a single patient's anonymized information.

## Consent

Informed consent was obtained from the patient's caregivers specifying the purpose of the article and the submitted figures.

## Conflicts of Interest

The authors declare no conflicts of interest.

## Data Availability

Data sharing not applicable to this article as no datasets were generated or analysed during the current study.

## References

[1] L. Arash‐Kaps , K. Komlosi , M. Seegräber , et al., “The Clinical and Molecular Spectrum of GM1 Gangliosidosis,” Journal of Pediatrics 215 (2019): 152–157.e3. 31761138 10.1016/j.jpeds.2019.08.016

[2] F. M. Lang , P. Korner , M. Harnett , A. Karunakara , and C. J. Tifft , “The Natural History of Type 1 Infantile GM1 Gangliosidosis: A Literature‐Based Meta‐Analysis,” Molecular Genetics and Metabolism 129 (2020): 228–235. 31937438 10.1016/j.ymgme.2019.12.012 PMC7093236

[3] A. K. Rha , A. S. Maguire , and D. R. Martin , “GM1 Gangliosidosis: Mechanisms and Management,” Application of Clinical Genetics 14 (2021): 209–233. 33859490 10.2147/TACG.S206076 PMC8044076

[4] S. Minisola , C. Cipriani , L. Colangelo , G. Labbadia , J. Pepe , and P. Magnusson , “Diagnostic Approach to Abnormal Alkaline Phosphatase Value,” Mayo Clinic Proceedings 100, no. 4 (2025): 712–728. 40019430 10.1016/j.mayocp.2024.11.019

[5] G. Cannalire , S. Pilloni , S. Esposito , G. Biasucci , A. Di Franco , and M. E. Street , “Alkaline Phosphatase in Clinical Practice in Childhood: Focus on Rickets,” Front Endocrinol (Lausanne) 14 (2023): 1111445. 36817604 10.3389/fendo.2023.1111445 PMC9931734

[6] M. W. Ho and J. S. O'Brien , “Differential Effect of Chloride Ions on ‐Galactosidase Isoenzymes: A Method for Separate Assay,” Clinica Chimica Acta 32, no. 3 (1971): 443–450. 10.1016/0009-8981(71)90446-3 5096955

[7] V. Shkalim Zemer , M. Hoshen , Y. Levinsky , et al., “Benign Transient Hyperphosphatasemia in Infants and Children: A Retrospective Database Study,” European Journal of Pediatrics 182 (2023): 3211–3216. 37127797 10.1007/s00431-023-04995-1 PMC10151212

[8] B. M. Mogilner , Y. Barak , M. Amitay , and J. Zlotogora , “Hyperphosphatasemia in Infantile GM1 Gangliosidosis: Possible Association With Microscopic Bone Marrow Osteoblastosis,” Journal of Pediatrics 117 (1990): 758–761. 2135166 10.1016/s0022-3476(05)83338-4

[9] R. Denis , J. L. Wayemberg , M. Vermeulen , F. Gorus , I. Liebaers , and E. Vamos , “Hyperphosphatasemia in GM1 Gangliosidosis,” Journal of Pediatrics 120 (1992): 164. 1731018 10.1016/s0022-3476(05)80630-4

[10] A. Caciotti , S. C. Garman , Y. Rivera‐Colón , et al., “GM1 Gangliosidosis and Morquio B Disease: An Update on Genetic Alterations and Clinical Findings,” Biochimica et Biophysica Acta 1812 (2011): 782–790. 21497194 10.1016/j.bbadis.2011.03.018 PMC3210552

[11] I. Menkovic , M. Williams , N. Makhijani , et al., “Persistent Elevations of Alkaline Phosphatase as an Early Indicator of GM1 Gangliosidosis,” Molecular Genetics and Metabolism Reports 42 (2025): 101191. 39897471 10.1016/j.ymgmr.2025.101191 PMC11786200

[12] J. R. Jarnes Utz , S. Kim , K. King , et al., “Infantile Gangliosidoses: Mapping a Timeline of Clinical Changes,” Molecular Genetics and Metabolism 121 (2017): 170–179. 28476546 10.1016/j.ymgme.2017.04.011 PMC5727905

[13] C. R. Ferreira , D. S. Regier , R. Yoon , et al., “The Skeletal Phenotype of Intermediate GM1 Gangliosidosis: Clinical, Radiographic and Densitometric Features, and Implications for Clinical Monitoring and Intervention,” Bone 131 (2020): 115142. 31704340 10.1016/j.bone.2019.115142 PMC6937522

[14] Y. Ago , E. Rintz , K. S. Musini , Z. Ma , and S. Tomatsu , “Molecular Mechanisms in Pathophysiology of Mucopolysaccharidosis and Prospects for Innovative Therapy,” International Journal of Molecular Sciences 25, no. 2 (2024): 1113. 38256186 10.3390/ijms25021113 PMC10816168

[15] H. B. Al‐Kouatly , L. Felder , M. M. Makhamreh , et al., “Lysosomal Storage Disease Spectrum in Nonimmune Hydrops Fetalis: A Retrospective Case Control Study,” Prenatal Diagnosis 40 (2020): 738–745. 32134517 10.1002/pd.5678 PMC7260084

[16] H. L. Gray‐Edwards , D. S. Regier , J. L. Shirley , et al., “Novel Biomarkers of Human GM1 Gangliosidosis Reflect the Clinical Efficacy of Gene Therapy in a Feline Model,” Molecular Therapy 25 (2017): 892–903. 28236574 10.1016/j.ymthe.2017.01.009 PMC5383552

[17] M. T. Fiorenza , E. Moro , and R. P. Erickson , “The Pathogenesis of Lysosomal Storage Disorders: Beyond the Engorgement of Lysosomes to Abnormal Development and Neuroinflammation,” Human Molecular Genetics 27, no. R2 (2018): R119–R129. 29718288 10.1093/hmg/ddy155

[18] E.‐R. Nicoli , I. Annunziata , A. d'Azzo , F. M. Platt , C. J. Tifft , and K. M. Stepien , “GM1 Gangliosidosis—A Mini‐Review,” Frontiers in Genetics 12 (2021): 734878. 34539759 10.3389/fgene.2021.734878 PMC8446533

